# Keeping Low Reproductive Number Despite the Rebound Population Mobility in Korea, a Country Never under Lockdown during the COVID-19 Pandemic

**DOI:** 10.3390/ijerph17249551

**Published:** 2020-12-20

**Authors:** Soyoung Kim, Yae-Jean Kim, Kyong Ran Peck, Youngsuk Ko, Jonggul Lee, Eunok Jung

**Affiliations:** 1 Department of Mathematics, Konkuk University, Seoul 05029, Korea; soykim211@gmail.com (S.K.); kys1992@konkuk.ac.kr (Y.K.); 2 Department of Pediatrics, Samsung Medical Center, Sungkyunkwan University School of Medicine, Seoul 06351, Korea; yaejeankim@skku.edu; 3 Division of Infectious Diseases, Department of Medicine, Samsung Medical Center, Sungkyunkwan University School of Medicine, Seoul 06351, Korea; krpeck@skku.edu; 4 INSERM, Pierre Louis Institute of Epidemiology and Public Health, Sorbonne Université, 75646 Paris, France; jg4lee@gmail.com

**Keywords:** COVID-19, mathematical modeling, reproductive number, population mobility, lockdown, Republic of Korea

## Abstract

Nonpharmaceutical intervention has been one of the most important strategies to prevent the spread of the SARS-CoV-2 in the communities during the COVID-19 pandemic. Korea has a unique experience that we had the first large outbreak during the early pandemic and could flatten the epidemic curve without lockdown. In this study, the effective reproductive numbers were calculated for the entire nation and Seoul (the capital city) Metropolitan Area from February 16–15 July, where 60% of the population reside. We compared the changes in population mobility data and reproductive number trends according to the changes in the government’s nonpharmaceutical intervention strategy. The total daily mobility decreased when Korea had the first wave of a large outbreak in February–March 2020, which was mainly caused by the decrease of daily noncommuting mobility. However, daily commuting mobility from 16 February to 30 June 2020 was maintained at a similar level since there was no national lockdown for workers who commute between home and work. During the first half-year of 2020, Korea could control the outbreak to a manageable level without a significant decrease in daily public mobility. However, it may be only possible when the public follows personal hygiene principles and social distancing without crisis fatigue or reduced compliance.

## 1. Introduction

Since unidentified pneumonia cases were reported to the World Health Organization (WHO) by China in December 2019, a novel infectious virus, severe acute respiratory syndrome coronavirus-2 (SARS-CoV-2), has spread globally [1]. The Republic of Korea is the third country where a patient with coronavirus disease 2019 (COVID-19) was identified outside of China [2]. With intensive contact tracing and active quarantine policies, the epidemic seemed to be totally controlled to prevent local transmission. However, on 16 February, a case without travel history and any epidemiological link was reported, and the authorities were warned of community transmission [3,4]. On 23 February, the Korea Center for Disease Control and Prevention (KCDC) raised the COVID-19 alert to the highest level of “red” to strengthen the overall response system [5]. Since the first patient was diagnosed on 20 January 2020, the number of COVID-19 cases escalated during the first large outbreak in February and March. During this time, approximately 400 confirmed cases were reported every day [6,7]. Korea ranked second globally in terms of the number of confirmed cases, following China at that time [8]. The KCDC continued rapid and massive contact tracing and confirmation tests. At the same time, nonpharmaceutical interventions (NPI) were implemented to mitigate COVID-19 spread. Through enhanced social distancing policies and regulation and rapid confirmation processes, the number of daily cases declined to less than 30 by 20 April [9]. After social distancing was eased, the disease has been still spreading in society without a surge [10].

As the COVID-19 vaccine and effective antiviral drugs have not been discovered, the only intervention is an NPI strategy, including social distancing [11]. The NPIs, such as social distancing, contact tracing, and quarantine of close contacts, are critical for flattening the epidemic curve in the early phase of the outbreak. Many countries closed their borders, strongly restricted the movement of individuals, and implemented a lockdown policy. These control strategies succeeded in the societal transition from the increasing to decreasing phase of the epidemic. However, after the lockdown eased, the number of COVID-19 cases increased again and, in some countries, more COVID-19 cases were confirmed than that before the lockdown [12]. Korea never had a lockdown; the government only implemented varying levels of social distancing and rigorous contact tracing with fast testing, which have been successfully flattening the epidemic curve when there were major surges in February and March 2020 and additional subsequent outbreaks [12].

Mathematical modeling is beneficial for analyzing the transmission dynamics of infectious diseases [13]. With the reported data, the transmission rate, which is hardly measurable, can be estimated. Using the estimated transmission rate, the reproductive number can be calculated. This study aimed to analyze the COVID-19 epidemic in Korea using mathematical modeling. In this study, we addressed the changes in population mobility data and trends of the reproductive number were compared and analyzed according to the changes in the government’s NPI strategy.

## 2. Materials and Methods

### 2.1. COVID-19 Epidemic in the Republic of Korea

Since the first COVID-19 case was confirmed on 20 January 2020, every individual in close contact with a confirmed case was screened and quarantined to prevent local transmission. At that time, all confirmed cases were those who had entered Korea from other countries or were in close contact with previously confirmed cases. Since 16 February, the locally transmitted cases were continuously discovered, especially in Daegu and Gyeongsangbuk-do provinces. The Korean government has been implementing social distancing strategies (Table 1) in several stages, considering the number of confirmed cases and economic situations. On 29 February, the KCDC recommended the public practice of social distancing until the beginning of March (level II). The social distancing level was raised to level III from 22 March until 20 April. Social distancing policy level III, which restricts daily socioeconomic activities, was not sustainable for the long term and affected the economy. Social distancing level III was terminated on 20 April and lowered to level I, allowing the usual daily socioeconomic activities from 6 May.

**Figure 1:**
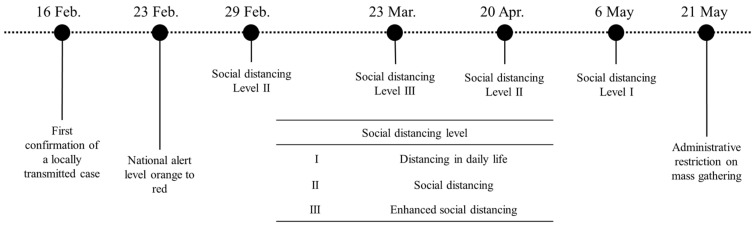
Timeline of significant events for COVID-19 local transmission.

As the social distancing was eased, community transmission increased again, mainly around crowded entertainment facilities in Seoul Metropolitan Area (SMA). Mass gathering at the high-risk facilities was restricted from 21 May. Figure 1 summarizes the timeline of significant events applied in this study. Detailed NPIs according to the social distancing level are listed in Table 1.

### 2.2. Mobility Data Analysis

The public mobility was measured using statistical population data provided by KT, one of the big three mobile communication company with a Korean market share of 30%. We aggregated the number of population movements regardless of the regions, ages, and sexes. Taking the average for each period, the aggregated number of population movement is converted to ten levels and compared with the reproductive number. The public mobility in the Seoul Metropolitan Area (SMA) was analyzed separately. Commuting was defined as movement to a place, which was estimated to be the weekly residence of each population; otherwise, the mobility was classified as noncommuting. The mobility data were collected from 16 February to 30 June.

### 2.3. Mathematical Model

**Figure 2:**
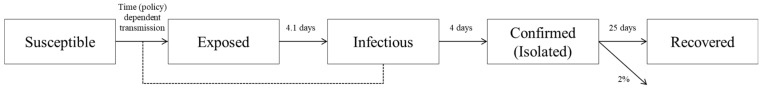
Flow diagram of COVID-19 transmission dynamics.

The COVID-19 transmission dynamics is described with a mathematical compartment model. This model consists of ordinary differential equations representing the movement of each compartment (see Supplementary Materials). In this work, the total population is divided into five classes: susceptible, exposed to the virus, infectious to susceptible individuals, laboratory-confirmed and isolated, and recovered. SARS-CoV-2 can be transmitted from an infectious to a susceptible individual. Once a susceptible individual is exposed, he/she becomes infectious after an incubation period of the virus. The infectious individuals are isolated after the laboratory confirmation test. Finally, isolated patients are discharged when they recover. Figure 2 describes the flow of COVID-19 transmission dynamics. The arrows represent the epidemic progression in the population. The dotted line indicates the possibility of transmission from infectious to susceptible groups. The numbers next to the arrows represent the averaged value of the period to move to the next compartment or fatality rate.

It is known that the incubation period of SARS-CoV-2 is 1–14 days [14]. According to the KCDC report, the average incubation period is set to 4.1 days [15]. Thus, the rate of progression from exposure to infectious individuals is assumed to be 1/4.1 days^−1^. The average period of moving from an infectious to a confirmed case is 4 days; the rate of confirmation is 1/4 days^−1^ [16]. It is assumed that the confirmed cases are totally isolated and would not infect others. The isolated patients are discharged after an average of 25 days. In other words, the averaged recovery rate is 1/25 days^−1^ in this model. As of 15 July 2020, 289 cases were deceased among 13,551 confirmed cases. The fatality rate is approximately 2%.

The transmission rate of COVID-19 is estimated by minimizing the residual between the model curve and reported data. The least-squares fitting optimization tool, *lsqcurvefit*, in MATLAB, is used to estimate the best-fitted parameters. The daily cumulative confirmed cases from 16 February to 15 July were used, and the phase is divided into seven consecutive periods according to the government intervention policies, as shown in Figure 1. Note that to describe the local transmission of the COVID-19, the imported confirmed cases in other countries are not included in this work.

## 3. Results

### 3.1. Daily Population Mobility Data

**Figure 3:**
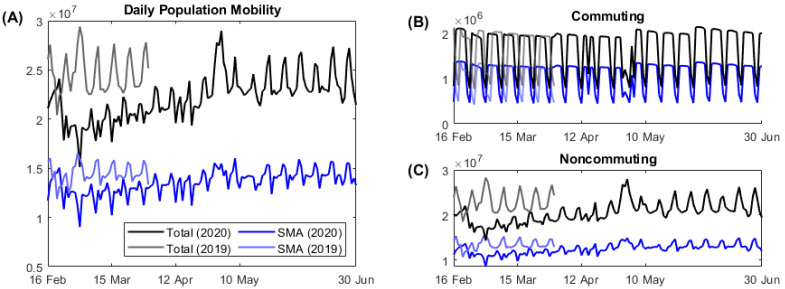
Daily population mobility data from 16 February to 30 June. (**A**) Total population mobility; (**B**) daily commuting; (**C**) daily noncommuting.

On average, the population mobility in SMA accounts for approximately 60% of the mobility at the nationwide level. Compared to the same period in 2019, the population movement decreased, especially noncommuting movements, in the early period of the COVID-19 outbreak (Figure 3). By the end of February, when the number of daily COVID-19 cases approached 1000, the noncommuting population reduced by 25%. Since then, public mobility gradually increased later despite the implication of social distancing level Ⅱ by the governmental policies. On 15 April, the national election day, commuting reduced to the level of weekends and noncommuting increased. During the spring holiday season in 2020 (from 30 April to 5 May), daily population mobility reached maximum because the noncommuting population increased significantly.

### 3.2. Reproductive Number

**Figure 4:**
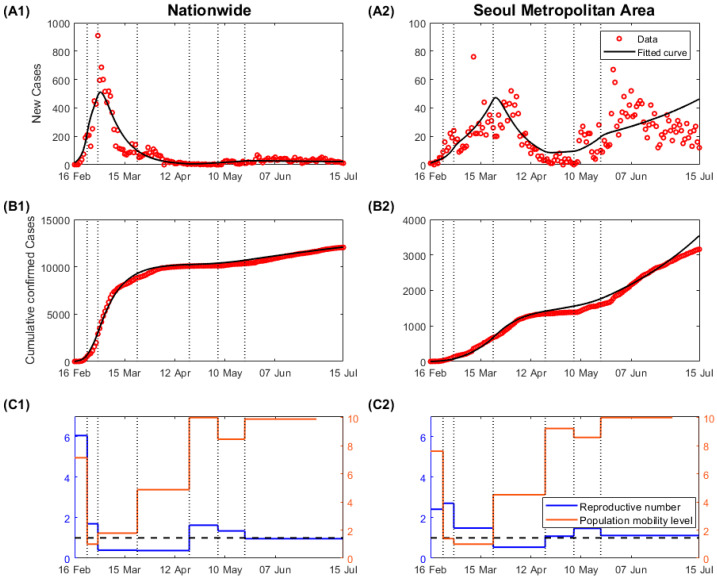
Data fitting results and projections, the reproductive number and population mobility levels according to the social distancing level: (**A**) daily confirmed cases; (**B**) cumulative confirmed cases; (**C**) reproductive number and population mobility level.

Figure 4A,B shows daily and cumulative confirmed cases from 16 February to 15 July in nationwide and SMA, respectively. In Korea, most of the population is concentrated in SMA, where approximately half of the total population lives. At the same time, this area covers only 11.8% of the country’s land area. The high population density makes it conducive to the spread of infectious diseases. However, the transmission of the virus in SMA was not a major issue in the early phase of the epidemic, given the relatively fewer cases compared to those in other regions. Most of the cases in the early phase of the epidemic occurred in Daegu and Gyeongsangbuk-do provinces, in connection with a religious mass gathering in extremely crowded spaces. As a result of intensive contact tracing and a quarantine system, the epidemic curve could be flattened within two months. From a national point of view, the massive epidemic, as seen in February, is not recurring. Figure 4 shows that the disease spreading pattern in SMA was different from that at the national level. As the first clustered cases from Daegu had been contained, most of the new cases were reported in SMA, which became a transmission source for the epidemic. Due to high population density and complexity, the possibility of disease outbreaks is higher in SMA. It was difficult to screen all contacts, and the transmitted cases despite contact tracing were a result of secondary cases in other places. The disease spread more rapidly, and clustered cases were reported continuously.

The estimated transmission rates in each phase are listed in Table 2. As the number of community transmission cases increased significantly, the public became aware of the outbreak, and the transmission rate in nationwide decreased before the social distancing policies were officially put in place. The transmission rate further decreased when social distancing levels Ⅱ and Ⅲ were enforced. After reducing the social distancing level from Ⅲ to Ⅱ on 20 April, the transmission rate increased again, and the reproductive number became higher than 1.0. Following the regulation of high-risk facilities on 21 May, the transmission rate decreased.

The reproductive number of an infectious disease indicates the number of secondary cases from a single infectious individual during an infectious period. This is calculated by multiplying the transmission rate by the infectious period, which is four days in this study. The reproductive number in each phase is depicted in Figure 4C as blue lines. The black horizontal dashed line represents the reproductive number, which equals 1. Given the relatively lower cases in SMA, the transmission rate and the corresponding reproductive number were lower in the early phase. Given that the COVID-19 in South Korea was occurred due to mass attendance in extremely crowded places, which led to a nationwide spread, the transmission rate in SMA may represent an appropriate epidemic risk in the country. At the beginning of the epidemic, the reproductive number was approximately 2.4, which was similar to that reported by the WHO (2–2.5) [17]. The reproductive number decreased with the implementation of the social distancing policy, and it increased after the social distancing level was eased. After 21 May, most cases have been reported in SMA, and the reproductive number in SMA (1.12) is higher than the national average (0.96).

### 3.3. Comparison between Population Mobility and Reproductive Number

After the SARS-CoV-2 was transmitted in society, the movement decreased significantly, and the reproductive number also decreased. However, the public movement increased over time despite the implication of social distancing policies mandated by health authorities. Despite the increased population mobility, because of the enhanced social distancing interventions from 23 March to 20 April (Table 1), the reproductive number remained low and further decreased in SMA. This may be because the government prohibited mass gatherings in crowded places, and the public maintained adequate personal hygiene levels, including wearing face masks compulsorily.

## 4. Discussion

In this study, we investigated the impact of population mobility using mobile communication signal data on social distancing level. Mathematical modeling was used to calculate the reproductive number in Korea, a country that never closed its borders or implemented a nationwide lockdown but only enforced various levels of social distancing strategies. We found that during the early phase of the COVID-19 outbreak surge in February 2020, population mobility was high. The resulting reproductive number was also high, but it dropped after implementing a social distancing policy. According to a survey from 4 February to 2 April, approximately 90% of the respondents reported practicing social distancing activities such as avoiding outdoor activities, public transportation, and healthcare facilities [18]. An interesting observation is that from 23 March to 20 April, the period of social distancing level Ⅲ, the reproductive number was still less than 1, although the population mobility continued to increase. Furthermore, even with the eased social distancing level and the apparent increase in population mobility after mid-April, the reproductive numbers remained low.

Reduction in public mobility can decrease the level of local transmission. It must be noted that the reproductive number was not increased significantly, despite an increase in public mobility during the early period of COVID-19 pandemic in Korea. Enhancing personal hygiene levels, regulation of high-risk facilities, and disinfection can reduce the transmission even when personal movement is increased. It is reported that approximately 80% of the population enhanced their personal hygiene by wearing a mask and washing hands more frequently. This is a significant improvement compared to that observed during the 2015 MERS outbreak (wearing mask 15.5%, washing hands 60.3%) [18].

During an early period of the COVID-19 pandemic in February–March 2020, a reduction in daily public mobility (especially noncommuting mobility) in Korea could have contributed to the outbreak control. However, as the first large outbreak became under control at a manageable level in May–June, the public mobility increased. Of interest, this did not lead to an additional large outbreak by 30 June 2020. We assume that although the mobility increased, personal hygiene measures were more widely implemented as a part of daily routines with easier access to mask purchase, stricter mask-wearing policy (e.g., universal mask-wearing policy in the subway), and hand hygiene. In addition, the effect of a high uncertainty avoidance index among Koreans could have a role in maintaining the low transmission despite the increased mobility during this first half of the year before the general public became fatigued with social distancing [19].

The COVID-19 pandemic is ongoing, and numerous cases are occurring around the world. As a result of the government’s preemptive response and public cooperation, the epidemic curve could be flattened during the early epidemic in Korea. The transmission was identified in connection to a religious network, whereby the government could easily screen all high-risk individuals. This led to a rapid decrease in the number of daily cases. The nationwide reproductive number (averaged from 21 May to 15 July) is approximately 0.96. However, the problem is that most of the cases have been concentrated in SMA since May. In SMA alone, the reproductive number was approximately 1.12. The model warns that the transmission rate needs to be lower in SMA.

As the results of the mathematical model warned, Korea is currently experiencing the third wave of a large outbreak as of December 2020, mainly in SMA. This could be in some part due to crisis fatigue or social distance fatigue among Koreans. In other words, although they still follow the social distancing policies or personal hygiene at a high level among strangers or public areas because Koreans have high Uncertainty Avoidance Index, but they may not observe the rules properly with family/relatives or acquaintances.

This study has some limitations. The mathematical model assumes homogeneous mixing within the same group. However, the contact pattern can differ according to ages, occupations, and living area. These factors could be considered in future studies. We investigated the relationship between mobility and the reproductive number. There are numerous factors to consider, such as the time-dependent rate of wearing masks or conducting flexible working hours, but those are not reflected in this study. If more data becomes available, further analysis would be conducted.

## 5. Conclusions

The Korean government has never implemented a lockdown policy. For example, the national elections were held on 15 April. All individuals were required to keep their distance in a queue, wear a mask, sanitize their hands, and use a disposal globe to vote. With such efforts, no transmission cases during the voting period were reported. This highlights the fact that an increase in personal movement does not necessarily lead to disease spread if persons involved strictly comply with the social distancing measures.

The results of this study need to be interpreted with caution because NPIs were accompanied by intensive contact tracing, quarantine and testing even asymptomatic contacts. After a suspected case was confirmed, their close contacts, such as family members and colleagues, were required to be quarantined at home. The epidemiologic investigation was double-checked using surveillance cameras, mobile signaling, and credit card verification data. If a specific place was classified as a high-risk facility, the government encouraged individuals who previously visited that place to undergo a confirmation test and stay at home. Due to the national health insurance system, the cost of confirmation testing and hospitalization was not a personal burden in Korea. These contributed to shortening the infectious period and preventing secondary transmission. The number of isolated patients and the recent trends of the epidemic have been announced daily since January. The clarity of information helped the public to cooperate with the government’s strategies to mitigate disease spread. The South Korean response against the COVID-19 may not be applicable to other countries. However, it can serve as a good reference to implement NPIs to control COVID-19.

Reducing the number of contacts by mobility restriction may be an effective NPI but difficult to achieve in the long term. If the transmission is reduced by wearing a mask, enhancing personal hygiene, and controlling specific high-risk facilities, it is possible to minimize disease spread without mobility restriction [20].

In conclusion, during the first half-year of 2020, Korea could control the outbreak to a manageable level without a significant decrease in daily public mobility. However, it may be only possible when the public follows personal hygiene principles and social distancing without crisis fatigue or reduced compliance. We observed that mobility data seems to be a useful tool to understand the epidemiology and estimate the transmission rate in the community of COVID-19. However, it should be interpreted with the dynamic evolution of the viral epidemiology and behavioral changes of the public.

## Figures and Tables

**Table 1:** Applied interventions according to social distancing levels.

Interventions	Level I	Level II	Level III
Summary	Allowing daily socioeconomic activities as much as usual	Recommended to avoid going out, gatherings, and high-risk public facilities	Closing high-risk public facilities
Hand hygiene	Always	Always	Always
Wearing a facemask	Mandatory in public transports	Mandatory in public	Mandatory in public
Off sick	Recommended	Recommended	Recommended
Distancing	Avoid crowding and close contact	Avoid crowding and close contact	Avoid crowding and close contact
Indoor	Recommending regular ventilation	Recommending regular ventilation	Recommending regular ventilation
Workplace	Regular disinfection and ventilation	Encouraging working from home if possible	Encouraging working from home if possible
School	Partly opened	Closed	Closed
Mass gathering	Allowed, but recommended to avoid	Recommended to avoid gatherings	Not allowed

**Table 2:** Estimated transmission rates according to government’s social distancing level.

Region	16 Feb–22 Feb	23 Feb–28 Feb	29 Feb–22 Mar	23 Mar–19 Apr	20 Apr–5 May	6 May–20 May	21 May–15 Jul
Nationwide	1.5115	0.4235	0.0987	0.0946	0.4068	0.3358	0.2403
SMA ^1^	0.6038	0.6770	0.3717	0.1358	0.2717	0.3683	0.2795

^1^ SMA: Seoul Metropolitan Area.

## Supplementary Materials

The following are available online at https://www.mdpi.com/1660-4601/17/24/9551/s1, Document: Model Description.
