# Supplementary material for: Keeping Low Reproductive Number Despite the Rebound Population Mobility in Korea, a Country Never under Lockdown during the COVID-19 Pandemic
Source: Int J Environ Res Public Health. 2020 Dec 20;17(24):9551. doi: 10.3390/ijerph17249551 (PMC7765860; doi:10.3390/ijerph17249551)
Supplement: Supplementary file 1 [file ijerph-17-09551-s001.pdf]

Model Description

The COVID-19 transmission dynamics can be described by the following ordinary differential equations (ODEs). The total population (N) is divided in to five compartments: susceptible (S), exposed (E), infectious (I), confirmed and isolated (C), and recovered (R)

$$\frac{dS}{dt} = -\beta(t)\frac{SI}{N}$$

$$\frac{dE}{dt} = \beta(t)\frac{SI}{N} - \kappa E,$$

$$\frac{dI}{dt} = \kappa E - \alpha I,$$

$$\frac{dC}{dt} = \alpha I - \gamma C,$$

$$\frac{dR}{dt} = (1-f)\gamma C,$$

where $N = S + E + I + C + R$.

An individual in susceptible group is moved to the exposed group by transmission from the infectious individuals. The time dependent function $\beta(t)$ represents the transmission rate affected by the government policy. According to the social distancing level the transmission rate is changed. The number of transmitted individuals is represented by the term $\beta(t)\frac{SI}{N}$. The exposed individuals are moved to the infectious individuals. The parameter κ indicates rate of progression from exposed to infectious. The infectious individuals are confirmed and isolated. The confirmation rate (or isolation rate) is represented by a parameter α. The term αI indicates the daily number of reported cases and $\int_0^t \alpha I \, dt$ is the number of cumulated cases. The time dependent function $I(t)$ indicates the number of prevalence at time t. The infectious individuals are recovered at a rate of γ. The parameter f means fatality rate of the COVID-19 disease $(0 \leq f \leq 1)$.
